# Subcutaneous and Pulmonary Dirofilariasis with Evidence of Splenic Involvement

**DOI:** 10.1155/2016/8212387

**Published:** 2016-08-08

**Authors:** Adarsha Selvachandran, Raymond J. Foley

**Affiliations:** Division of Pulmonary/Critical Care, UConn Health, 263 Farmington Avenue, Farmington, CT 06030-1321, USA

## Abstract

Cases of human dirofilariasis have been reported in several countries around the world, including a large number in the Atlantic and Gulf Coast regions of the United States. Most commonly, these cases have subcutaneous or pulmonary involvement; however, there have been few reports of dirofilariasis involving structures such as large vessels, mesentery, the spermatic cord, and liver. We present a case of an unusual presentation of human dirofilariasis presenting as a shoulder abscess and what is presumed to be pulmonary and splenic involvement in a 55-year-old female.

## 1. Introduction

**Figure 1:**
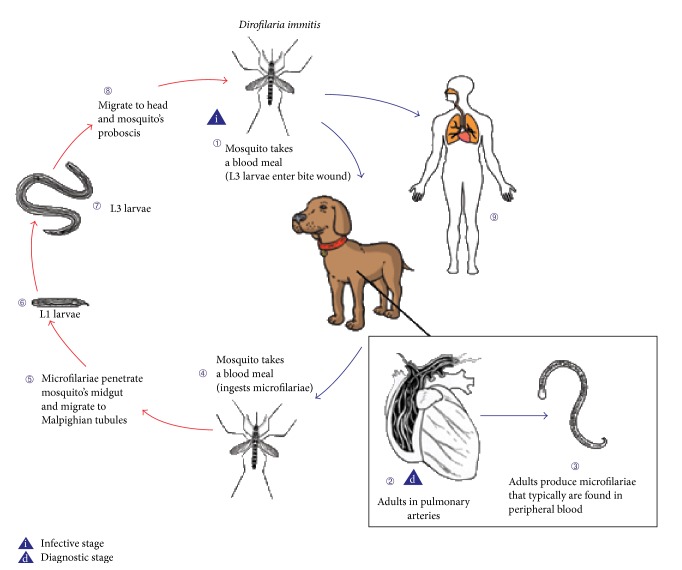
Biology-life cycle of* D. immitis* [1].

*Dirofilaria immitis*, colloquially known as the dog heartworm, is a nematode that utilizes dogs as its natural host. Humans serve as a dead-end host for* D. immitis*. In dogs,* D. immitis* exists as a mature worm in the right ventricle and pulmonary arteries. Cases of human dirofilariasis have been reported in several countries [1]. These case reports have described dirofilariasis involvement in multiple anatomic structures [2]. The mature female adult produces microfilariae that can be found in the dog's blood. When mosquitos feed on the dog, the microfilariae are ingested with the blood meal. The microfilariae then mature into larvae within the mosquito and ultimately migrate to the mosquito's proboscis. When the mosquito takes its next blood meal, it acts as a vector. The larvae leave the proboscis and enter the bite wound. When the vector mosquito takes a blood meal from a human, transmission of* D. immitis* to a dead-end human host would occur, provided the larva finds its way into the bite wound (see Figure 1) [3].

## 2. Case Presentation

**Figure 2:**
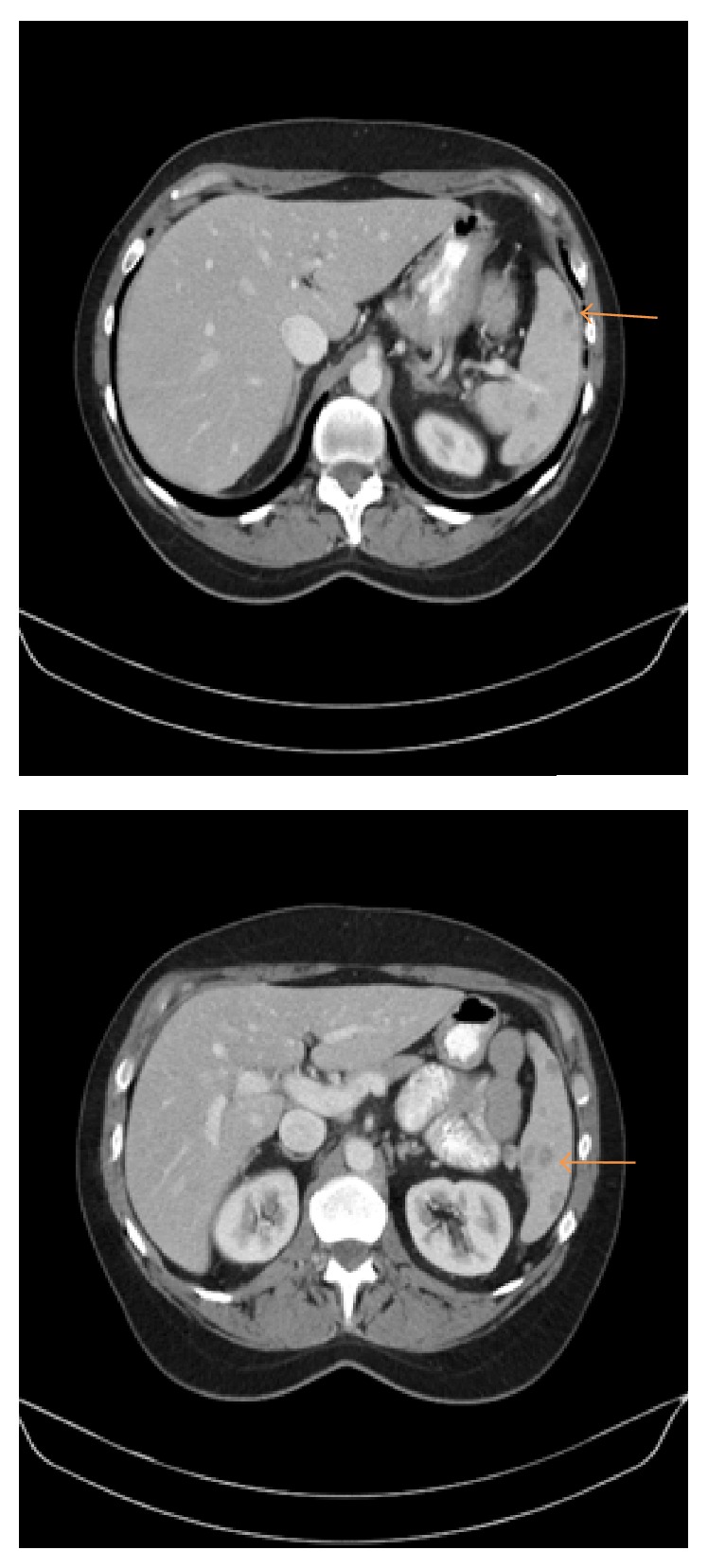
Abdominal imaging, revealing multiple splenic hypodensities.

**Figure 3:**
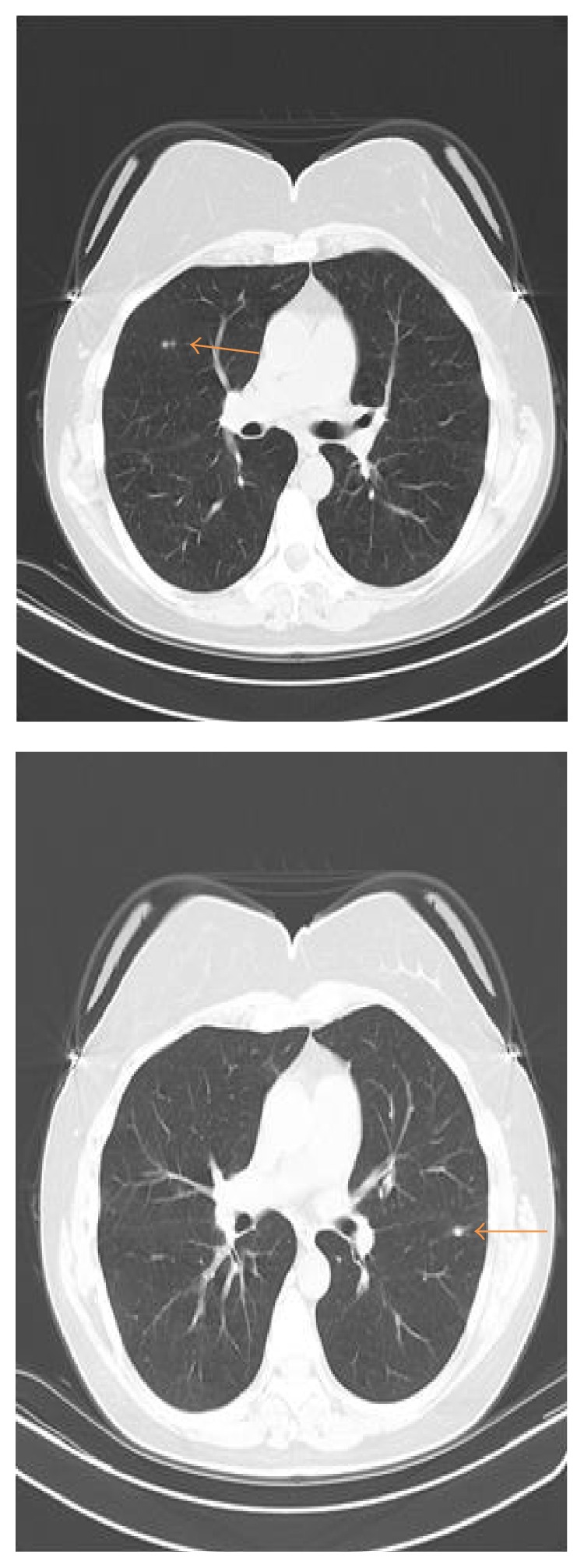
Chest CT scan, revealing bilateral nodules.

A 55-year-old female initially sought medical attention for abdominal pain following a hernia repair. On presentation, her physical exam and routine bloodwork (including a complete blood count and chemistry profile) were entirely normal. She subsequently underwent additional diagnostic testing. The abdominal computerized tomography (CT) scan demonstrated multiple splenic hypodensities along with a left lower lobe nodule (see Figure 2). For this reason, the patient subsequently underwent a dedicated chest CT scan for further evaluation. The chest CT scan revealed multiple nodules involving the right and left lung (see Figure 3). Based upon these imaging findings, our initial differential diagnosis included hemangiomas, lymphoma, fungal infection, sarcoidosis, splenic infarctions, and metastatic nodules. The patient's past medical history was positive for a surgical excision of a right shoulder mass 2 years earlier. The pathology report revealed evidence of* D. immitis* within the shoulder mass. Additionally, she was a lifetime nonsmoker who resided in Connecticut and had no exposure to dogs at home. Her family history was noncontributory.

After the acute episode of her abdominal pain resolved, the patient underwent additional evaluation. Her laboratory data showed that the liver function tests were within normal limits. Serum protein electrophoresis was within normal limits. Serologic testing including* Aspergillus* antigen,* Cryptococcus* antigen, and* Histoplasma* antigen was negative.

Bone marrow aspiration and biopsy showed normal cellular bone marrow with maturing trilineage hematopoiesis. There was no evidence of any granulomatous or lymphoproliferative processes.

A transthoracic echocardiogram showed normal left and right ventricular size and function. There were also findings of minimal tricuspid valve regurgitation and mitral valve thickening. A repeated transthoracic echocardiogram with bubble study did not reveal a patent foramen ovale (PFO) or atrial septal defect. Although not conclusively proven by a splenic biopsy, the cardiologist consultant's impression was that there was no cardiac cause for the presence of* D. immitis* in the splenic system. Follow-up chest, abdomen, and pelvis CT scan 6 months later showed stable to slightly decreased size and appearance of the splenic hypodensities and no evidence of new active disease in the abdomen or pelvis. Chest CT showed stable right and left nodules.

Following this extensive evaluation, the patient was deemed to be clinically and radiologically stable. A repeated chest CT scan was ordered 1 year later in order to ensure stability. The CT scan confirmed the stability of the pulmonary nodules with no changes to heart size and no evidence of mediastinal or hilar lymphadenopathy.

## 3. Discussion

The interesting aspect of this patient's presentation is the presence of the splenic hypodensities and multiple pulmonary nodules. Published reviews have shown that 75–95% of pulmonary dirofilariasis presents as a single granuloma, which was not the case in this patient [4]. As previously described,* D. immitis* normally enters the blood stream as larvae and migrates to the right ventricle where it develops into a sexually immature worm. When it dies, it is washed into the pulmonary artery and embolizes [5]. Typically the embolization of the deceased worm into the pulmonary arterial tree results in lesions at the lung periphery [6]. In this case, however, the splenic hypodensities indicate that the embolization of the immature adult worm somehow entered the systemic circulation rather than remaining isolated to the pulmonary circulation. The cause of this unusual finding has yet to be reported in the medical literature.

The first explanation under consideration is a cardiac defect, which is why an echocardiogram was pursued. The thought process was that if the patient had PFO or a septal defect, there would be a venue through which an adult worm in the right ventricle could be embolized and enter both the pulmonary circulation and the systemic circulation to end up in the spleen. As reported, the patient had no evidence of such defect on color Doppler or on bubble study, making this explanation less likely. A second explanation could be that there was dissemination of the worm during surgical excision of the patient's right shoulder mass.

**Figure 4:**
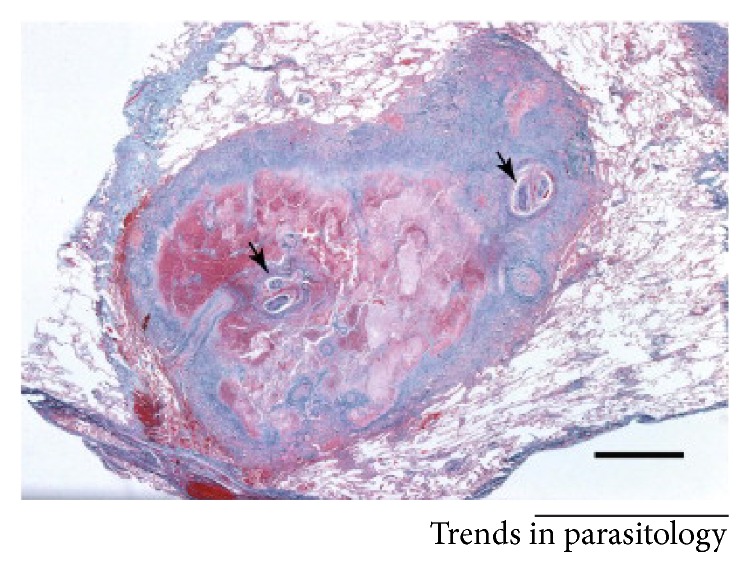
Courtesy of trends in parasitology: a coin lesion excised from a human lung showing a well-demarcated granuloma. Cross sections of the worm are highlighted by the black arrows [8].

Although in this case neither the splenic nor pulmonary lesions were biopsied, the stability of the lesions in conjunction with the patient's history of subcutaneous dirofilariasis makes dirofilariasis the most likely explanation. These lesions have been described in the literature to be well circumscribed and yellowish-grey in color, with normal lung parenchyma surrounding the lesion when biopsied. Microscopic pathology has been reported to show a central zone of necrosis, with a granulomatous zone with epithelial cells, plasma cells, lymphocytes, and a few scattered giant cells and an outer layer of fibrous tissue. Additionally, fragmented, necrotic portions of the embolized worm have been reported (see Figure 4) [7].

The most common symptom reported has been cough, with a lesser incidence of chest pain, fever, and hemoptysis. [7] In cases of human pulmonary dirofilariasis, medical treatment against the parasite is not indicated as the fragment of the worm has already been encapsulated by the immune system.

From a diagnostic perspective, video-assisted thoracoscopic biopsy is the most direct technique for diagnosing human pulmonary dirofilariasis as less invasive serologic testing is not currently available [4]. In the past, thoracotomy with wedge resection was employed in cases where malignancy was considered and needed to be ruled out.

**Figure 5:**
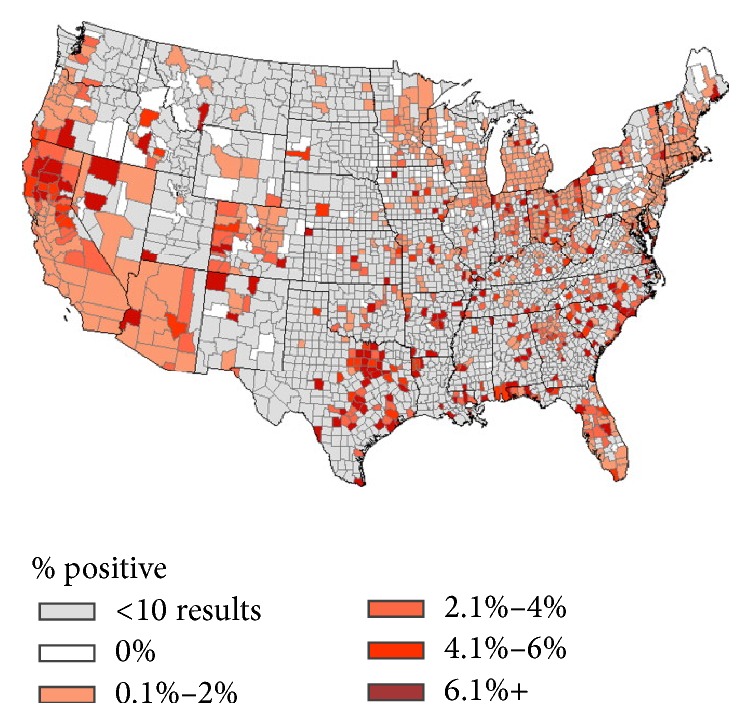
Courtesy of veterinary parasitology: US distribution of dogs testing positive for* D. immitis* antigen [8].

Risk factors for dirofilariasis in humans are dependent on the size of the dog population, the prevalence of canine dirofilariasis in the population (see Figure 5), and the density of vector mosquitos in the area. Most reported cases of human dirofilariasis are in areas where there is a high prevalence of canine dirofilariasis. As such, clinicians in these areas should be aware of the presence of dirofilariasis and entertain this entity on their differential diagnosis of pulmonary nodules. Awareness of dirofilariasis is important as it may decrease the amount of invasive testing and procedures the patient may undergo and thereby decrease healthcare-related costs.

As the patient was asymptomatic at follow-up, surgical intervention for these stable pulmonary and splenic lesions was deemed to be unnecessary (unlike the subcutaneous lesion in her right shoulder which required surgical resection for both diagnostic purposes and resolution of her pain).

A literature search for cases of human splenic dirofilariasis yielded no results to date. Although not conclusively proven, the splenic hypodensities can be assumed to be related to the presence of* Dirofilaria* in the splenic system. In the literature, other sites (including liver, lung, subcutaneous tissue, large vessels, peritoneal cavity, and spermatic cord) [2] have been reported.
